# Pendant breast immobilization and positioning in photoacoustic tomographic imaging

**DOI:** 10.1016/j.pacs.2020.100238

**Published:** 2020-12-26

**Authors:** Sjoukje M. Schoustra, Tim J.P.M. op 't Root, Rutger P. Pompe van Meerdervoort, Laurens Alink, Wiendelt Steenbergen, Srirang Manohar

**Affiliations:** a Multi-Modality Medical Imaging Group, Technical Medical Centre, University of Twente, P.O. Box 217, 7500 AE, Enschede, the Netherlands; b Biomedical Photonic Imaging Group, Technical Medical Centre, University of Twente, P.O. Box 217, 7500 AE, Enschede, the Netherlands; c PA Imaging R&D B.V., De Veldmaat 17, Bldg. 46/HTF-SEPA HI.222-224, 7522 NM, Enschede, the Netherlands

**Keywords:** Photoacoustics, Optoacoustics, Tomography, Breast imaging, Breast cancer, Breast support, Breast immobilization

## Abstract

This work describes the design, development and added value of breast-supporting cups to immobilize and position the pendant breast in photoacoustic tomographic imaging. We explain the considerations behind the choice of the material, the shape and sizes of a cup-shaped construct for supporting the breast in water in an imaging tank during full-breast imaging. We provide details of the fabrication, and other processing and testing procedures used. Various experiments were conducted to demonstrate the added value of using these cups. We show that breast movement during a measurement time of four minutes is reduced from maximum 2 mm to 0.1 mm by the use of cups. Further, the presence of the cup, centered in the aperture leading to the imaging tank, ensures that the breast can be reproducibly positioned at the center of the field-of-view of the detection aperture in the tank. Finally, since an accurate delineation of the water-tissue boundary can now be made, the use of the cup enables accurate application of a two-speed of sound model for reconstruction. All in all, we demonstrate that the use of cups to support the breast provides clear enhancement in contrast and resolution of breast images in photoacoustic imaging.

## 1 Introduction

In females, breast cancer is the most frequently diagnosed type of cancer worldwide [1]. Breast imaging has an important role in detection, diagnosis, monitoring, treatment and follow-up of breast cancer [2]. Currently used imaging techniques in the clinic include x-ray mammography (MMG), ultrasonography (US) and magnetic resonance imaging (MRI) [3]. MMG remains the only imaging technique sensitive enough to be used in screening programs, and has been proven to reduce breast cancer mortality [4,5]. However, it makes use of ionizing radiation and sensitivity of detecting breast cancer in women with high mammographic breast density is relatively low [6,7]. US does not require ionizing radiation, but is operator dependent [8]. MRI, a functional imaging method, is the most sensitive method for characterizing breast cancer [9]. Drawbacks include the need for contrast agent administration, high costs and a relatively low specificity [3,9]. Photoacoustic imaging (PAI), also referred to as optoacoustic imaging, is a promising technique being researched around the world for its potential role in different areas of breast cancer management [10]. The optical absorption contrast of tissue is exploited, where endogenous contrast is provided mostly by hemoglobin [11,12], but also lipid absorption might contribute to the obtained contrast [13]. This enables visualization of vascularization associated with cancer progression. Inducing angiogenesis (new blood vessel formation) is believed to be one of the biological capabilities of tumors and a hallmark of cancer [14]. Different imaging geometries for PA breast imaging are being developed and tested in clinical settings [10], ranging from handheld 2D linear [15,16] or curvilinear probes [13,17], planar two-dimensional (2D) systems [18,19], systems with a curved [20] or ring-shaped detector array [21,22], to systems with a hemispherical detector geometry [23,24].

Recently, we described our three-dimensional (3D) tomographic system for photoacoustic breast imaging: PAM 2 [25,26]. In this work, the first human subject measurements with the system were reported, where the breast was imaged while freely pendant inside the imaging tank filled with water. The measurements were prone to breast movements during the scanning time of four minutes, compromising the resolution and contrast of the images. Further, off-center positioning of the breast within the recording aperture often occurred, resulting in (part of) the breast not being imaged optimally. To counter these problems, it was decided to develop a breast-supporting cup to immobilize the breast, and so arranged as to position the breast in the center of the tank. The use of a breast cup would also lead to a fixed and known shape of the breast that would markedly ease the application of a two-speed of sound (2-SOS) reconstruction model, since the water-tissue boundary would be accurately known.

Other groups which perform 3D photoacoustic breast imaging with the subject in a prone position have described their approaches to support the breast. Lin et al. [22] used an agar pillow to slightly compress the breast against the chest wall, when the subject lies prone with the breast inside the water-filled imaging tank. Toi et al. [27] employed a spherically shaped breast-holding cup with a depth of 38 mm and a diameter of 240 mm, adapted from what was described in more detail by Kruger et al. [28]. This cup is thermoformed from a 0.020” (∼ 0.5 mm) thick sheet of polyethylene terephthalate (PETG). Acoustic coupling between breast and cup is ensured by placing water in the cup. Oraevsky et al. [23] reported on a hemispherical, optically clear, acoustically thin plastic cup-stabilizer and the use of an acoustic coupling medium.

In this work, we describe the design considerations behind the choice of the shape and sizes of breast-supporting cups, and details of the fabrication procedure used. We perform various imaging studies with the PAM 2 system on healthy human subjects, to investigate the added value of the use of the cups. Our results from raw signals from regions-of-interest show that the pendant unsupported breast undergoes slow settling movement in water to the extent of up to 2 mm during the scan of four minutes. Riding on this drift, we observe faster breathing movement patterns as well. In contrast, with the use of the supporting cups the breast movement is limited to ∼ 0.1 mm, and no breathing movement artifacts can be observed. The imaging results demonstrate that our approach enlarges the volume of the breast being measured, by positioning the breast in the center of the imaging tank. The use of the 2-SOS model for reconstruction with accurate delineation of the water-tissue boundary demonstrates clear enhancement in contrast and resolution of the images. All in all, we show that the use of breast-supporting cups substantially improves image quality in 3D photoacoustic breast imaging.

## 2 Materials and methods

### 2.1 Design demands

The cup should (i) reduce image artifacts caused by movement of the breast within the current measurement time of four minutes, (ii) position the breast in the center of the tank, and (iii) create a known water-tissue boundary to employ an accurate 2-SOS reconstruction algorithm. The cup material and its thickness should be so chosen as to be both optically as well as acoustically (nearly) transparent, so that minimum signal degradation occurs. Ideally, the cup should support different sizes and shapes of breasts. Preferably, the use of the cup does not lead to discomfort or pain. Finally, it is of importance that the cup is so used as to ensure a hygienic situation for each measurement subject.

### 2.2 Cup design and fabrication

**Fig. 1:**
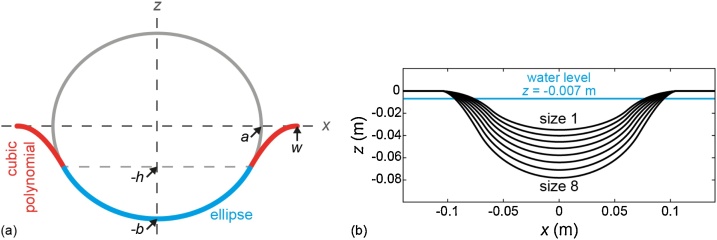
(a) 2D curve in *xz*-plane, consisting of an ellipse (blue) and a cubic polynomial (red). The cup is rotationally symmetric around the *z*-axis. The various geometrical parameters are explained in the main text. (b) 2D curves describing the eight designed cup sizes. The top curve corresponds to the smallest size (1) and the bottom curve to the largest size (8). The blue line designates the water level.

The shape of the cup is described by a 2D curve in the *xz*-plane (see Fig. 1), which is revolved around the *z*-axis to obtain a 3D structure. This rotational symmetry was chosen for practical purposes: ease of fabrication and positioning inside the imaging tank. But also because it is not possible to take into account all different possible breast shapes and personalize the cup shape accordingly. To accommodate breasts of different sizes, eight different sized cups were designed, following the same shape, but each with their own set of parameters defining the shape.

The base of the cup consists of an ellipse with long axis *a* and short axis *b* along the *x* and *z* directions respectively (Fig. 1(a)). At distance *h* (equal to *b*/2) from the center along the *z*-axis, the ellipse is connected to a cubic polynomial, from now on referred to as the flare. This flare or rounded edge serves to enhance comfort for the subject as well as to naturally guide the breast into the shape of the cup. The parameter *s* is the derivative of the curve at position *w* along the *x*-axis. The value of *s* is slightly off zero for all cups, to enhance firmness in vertical direction. The angle is kept small however, to create a smooth transition between cup and bed surface. The radius of the cup at *z* = 0 (where the cup touches the chest wall) is *w*. For each cup size, parameter *w* is equal to 0.105 m. Although in general, the diameter of the breast at the chest wall increases with breast size [29], we know from experience with human subject measurements that there are also smaller breasts with a relatively large diameter. Since we do not want to exclude these women, a fixed, relatively large value for *w* was chosen. For breasts with smaller diameters, it will not be a problem if the breast is not completely filling the cup, since the remainder of the cup is filled with water. Breast diameters at the chest wall did not exceed 19 cm in the research by Huang et al. [29], therefore we expect our cup diameter of 21 cm to be adequate for most women.

The maximum depth *b* for each size is listed in Table 1. The volume of the breast inside each cup was calculated, where the so-called submerged volumes per size are given in Table 1. Submerged volume refers to the part of the breast immersed in water and thus within the measurement volume of the system. A small distance of 7 mm between bed surface and water level was taken into account. See Fig. 1(b) for the 2D curves showing all eight cup sizes.

**Table 1:** Per cup size: maximum depth *b* (mm) and submerged volume (mL).

Size	Max depth *b* (mm)	Submerged volume (mL)
1	35	264
2	40	331
3	46	408
4	52	496
5	58	599
6	64	717
7	71	856
8	78	1013

**Fig. 2:**
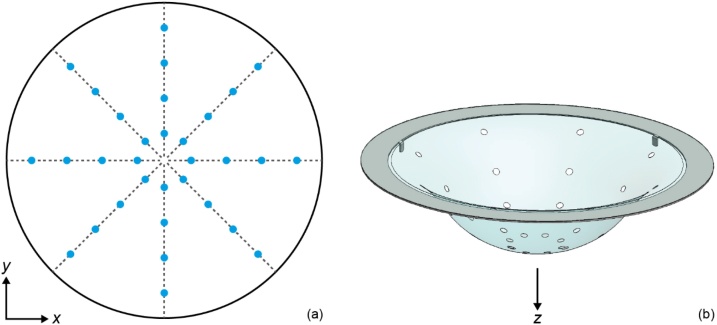
(a) Top-view sketch of a cup and the pattern of 32 holes (5 mm diameter) for water coupling. (b) 3D rendering of a cup with holes, mounted on a stainless steel ring.

Given the generic and rotationally symmetrical shape of the cup, it is expected that it will not perfectly fit all breasts with a probability of space being left between breast and cup. To ensure acoustic coupling between the breast and the cup in this eventuality, a multitude of holes are made into each cup to let water inside the cup from the imaging tank. This approach was chosen to ensure that there is enough water between breast and cup, and at the same time prevent water from spilling over the lip of the cup and onto the bed. Thirty two (32) holes, each with diameter 5 mm, are punched in an outward direction in a cup, to prevent potentially sharp edges of PVC from damaging the skin of the breast. See Fig. 2(a) for a top-view sketch of the pattern of holes, oriented along four axes.

The material used to fabricate the breast immobilizing cups is virgin polyvinyl chloride (PVC) sheet material with a thickness of 180 μm (Krumbeck Kunststoffverarbeitung GmbH, Stadtlohn, Germany). The PVC is a food grade material (according to EG nr. 1935/2004 and EU nr. 10/2011), warranting safe contact between the material and the skin. The material was tested to have an optical transmission >98 % in water, for both wavelengths (UV-2600 spectrophotometer with integrating sphere attachment ISR-2600Plus, Shimadzu Corporation, Kyoto, Japan). Acoustic transmission was measured at three frequencies: 0.5 MHz, 1 MHz and 1.5 MHz. Ultrasound transducers (V318 and V303 from Olympus NDT, Inc., Waltham, Massachusetts) were driven with an AFG3102 function generator (Tektronix, Inc., Beaverton, Oregon) and signals were amplified with an RF power amplifier (A075, Electronics & Innovation, Ltd., Rochester, New York). Transmitted signals were detected with a fibre-optic hydrophone system (FP197-24T, Precision Acoustics, Ltd., Dorchester, UK). Transmission through the cup material was compared to transmission through water alone. Average transmission losses were calculated to be: 9.1·10^−2^ dB at 0.5 MHz, 8.5·10^−1^ dB at 1 MHz and 1.2 dB at 1.5 MHz.

For each size cup, a mold was 3D-printed from nylon by means of selective laser sintering. Each mold was smoothened by coating the surface with a regular paint primer and polishing it with sanding paper in a water bath. A vacuum shaping machine (Formech 300, Formech International Limited, Hertfordshire, UK) was used to heat and shape the PVC sheet material into cups. Thicknesses throughout the cups were affected by this shaping process. Thicknesses of around 160 μm were measured at the flare and center of the cup, where the average thickness in between was approximately 150 μm, also depending on the cup size (thinner for larger cups). The flare of the cups extends into a rim at *z* = 0, which lies on a stainless steel ring mounted in the aperture of the PAM 2 bed. Fig. 2(b) shows a 3D rendering of one of the cups with holes, mounted on such a stainless steel ring.

### 2.3 Healthy subject imaging

The performance of the supporting cups was tested by imaging healthy volunteers with PAM 2. A detailed description of the PAM 2 system can be found in Ref. [25]. The measurement protocol was reviewed and approved by an Institutional Review Board (METC Twente, Medisch Spectrum Twente, Enschede, The Netherlands). All subjects were informed and fully competent to give consent. Only post-menopausal women above the age of 50 were measured. The expectation was that measurements on their relatively fatty breasts would benefit more from the supporting cups than the more dense and firm breasts of younger women. Moreover, the majority of breast cancer patients is above 50, reflected in the starting age of 50 for breast cancer screening in the Netherlands. Subjects with symptoms of breast cancer, a history of (benign or malignant) breast lesions, a history of surgery or radiation therapy on the breasts, bloody discharge, ulcers or wounds on the breast were excluded from the study. Also, subjects who had undergone a breast biopsy in the 6 months prior to the study, and subjects undergoing chemotherapy at the time of the study were excluded.

Prior to imaging a subject, one of her breasts was fitted by the system operator into different sized cups in a forward leaning position. Hereby, an estimation was made of the best fitting cup size. Subsequently, a tomographic scan of the subject’s right breast, supported with the chosen size (*n*) cup, was performed. The right breast was suggested by the operator, but each subject was free to have the left breast imaged. All subjects chose to have the right breast imaged. Two more tomographic scans were obtained of the right breast; one with a cup one size-step smaller (*n* – 1) and one with a cup one size-step larger (*n* + 1) than the chosen size *n*. A final scan was done without cup-support, with the breast directly pendant in the water-filled imaging tank.

For each scan, 45 projection angles (total covered angle of 60 deg) were measured, where 35 laser pulses per wavelength per average were taken at each angle. In one scan, two wavelengths pulsed in an interleaved manner, such that we obtained one dataset with 755 nm and one dataset with 1064 nm illumination. The wavelength of 1064 nm penetrates deep into tissue [30], but water absorption is relatively high too [31]. Excitation with 755 nm was chosen for its higher hemoglobin and lower water absorption [32]. The combination of these wavelengths should give us the future possibility of discriminating between contributions of oxy- and deoxyhemoglobin and hereby assessing tissue oxygen saturation. During all measurements, the temperature of the water inside the imaging tank was maintained at ∼ 25 °C. One tomographic scan took four minutes.

Age, self-reported bra size, chosen cup size *n* and additionally measured cup sizes *n* – 1 and *n* + 1 are listed in Table 2. Subject 3 only reported cup size, not band size. All included subjects had a light skin tone.

**Table 2:** Subjects: age, self-reported bra size, fitted cup size, additionally measured cups.

Subject	Age (years)	Self-reported bra size (European), band and cup	Fitted cup size	Additional scans obtained with
1	63	90E	Cup 7	Cup 6, cup 8, no cup
2	55	75A	Cup 2	Cup 1, cup 3, no cup
3	51	D	Cup 7	Cup 6, cup 8, no cup
4	59	75E	Cup 7	Cup 6, cup 8, no cup
5	65	75E	Cup 6	Cup 5, cup 7, no cup

Averaged signals of all tomographic scans were used for image reconstruction using an iterative algorithm applying regularized least squares optimization. The algorithm is built on an acoustic forward model that is based on Green's function [33,34] together with the adjoint operator. The adjoint is used to calculate the gradient while iteratively solving the least squares optimization problem. Regularization is based on a local smoothing operator. The relative sensitivity of all 384 detecting elements is taken into account. The elements are considered identical in the modelling of frequency response and acoustic directivity. For all measurements, reconstruction starts with filtered backprojection (based on Ref. [35]) followed by five iterations. Images resulting from the fifth iteration are shown. For all measurements, the decrease of the residual error strongly flattened already at the fifth iteration. Because of the tradeoff between computational time and additional image improvement, the choice was made to stop after five iterations.

In measurements where the breast was supported, a two-layer SOS is assumed for the reconstruction, with the two layers being water and breast tissue. The known shape of the cup facilitates identification of the boundary between the water and breast tissue. The SOS of the coupling water used in the backprojection was determined by the water temperature [36], measured for each tomographic scan. For all measurements, an SOS of 1496 m/s was employed (corresponding to a water temperature of 24.7 °C). The SOS of the breast tissue was empirically optimized in one of three scans for each individual subject. For this, images with different tissue-SOS were visually compared, where we looked at contrast, level of detail, imaging depth and the recognizable known structures of blood vessels. Subsequently, the chosen SOS was also set in the other two scans obtained with cups and it was checked whether it led to a similar improvement. It was to be expected that, per subject, one value for all measurements would suffice, since it is the same breast in each measurement. The 2-SOS reconstruction method was verified by measuring on a phantom consisting of absorbing threads, placed inside a cup filled with an alcohol-water mixture. For measurements without breast support, a value of 1496 m/s (corresponding to the SOS of the water) was employed for reconstructing the entire volume. For each subject, one measurement with cup was reconstructed with one SOS (of water) and compared to that same measurement employing two SOS. For subjects 1 through 4, fitted cup size *n* was used; for subject 5, cup size *n* – 1 was used. The 1-SOS and 2-SOS reconstructions for this analysis were all done using only a filtered backprojection algorithm.

Reconstructed volumes (0.35-mm or 0.4-mm grid size) of measurements using 755-nm illumination are visualized as maximum intensity projections (MIPs) in two planes: the coronal (*xy*) and sagittal (*xz*) plane. Since this study is not about assessing relative contributions of chromophores, we chose to show only images of one wavelength, for consistency. Per subject, measurements performed with different sizes cups are qualitatively compared with each other and with the measurement done without cup. Quality assessment through univariate quality metrics such as contrast was considered, but found to be not straightforward in interpretation and not necessarily helpful in describing differences between results.

### 2.4 Movement analysis

Two more measurements were performed on subjects 1 through 4 listed in Table 2. Photoacoustic data was acquired, with the imaging tank kept at one position for 250 s, comparable with the four-minute full tomographic scan. For each subject, this measurement was done once with fitted cup size *n*, and once without a supporting cup. All individual time signals were acquired (not only averaged signals). For each measurement, 5000 time signals were acquired; 2500 for each wavelength, in an interleaved manner.

In processing the acquired data, signals were clipped to a certain time window consisting of 1500 sample points (corresponding to a receiving time of 60 μs), to contain the first high signal peak arising from the superficial part of the breast. The first and the last time trace that were acquired (by the same detecting element) were compared. By calculating the cross-correlation of the first signal and shifted copies of the last signal, both signals were aligned. The required shifts (in number of samples) were documented for each receiving element (384 in total), for all eight measurements. In order to remove outliers from the analysis, the alignments with correlation coefficients with a value of 0.6 or higher were used for the analysis. Alignments with shifts over 100 samples were also removed from the analysis. Both thresholds were chosen empirically. Per subject, the measurements without and with cup are compared in histograms, visualizing the distribution of required shifts for signal alignment.

Further, progressive shifts per detecting element were calculated in time by aligning the second signal with the first, the third with the first, and so on, for all signals in the measurement set. In this way, the required shifts in the course of four minutes were calculated and visualized as a function of measurement time. This analysis was performed on the data acquired from two subjects (1 and 4). For both subjects, a detecting element was chosen for which all signal alignments had a high correlation coefficient (above 0.6). For subject 1, this was an element halfway (14 of 32, where element 1 is the top element) on one of the detector arrays. For subject 4, it was an element at the bottom of an array (30 of 32). See supplemental content of [25] for an illustration of a detector array.

### 2.5 Breast volume assessment

For subjects 1 through 4 described in Table 2, an estimation of the breast volume was made. The imaging tank is always filled to the top and excess water is drained. After positioning the free breast following a measurement with known cup (with known volume), the excess water was collected and its volume registered. The volume of the last measured cup and the amount of drained water when positioning the free breast add up to the volume of the free breast in the water. The volume of the freely pendant breast of each subject was compared to the volumes of the cups that were used to support her breast.

## 3 Results

### 3.1 Movement analysis

**Fig. 3:**
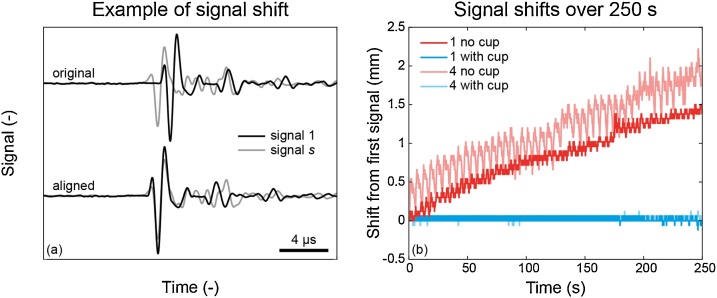
(a) Example of two signals before and after alignment. (b) Shift required for alignment of all 2500 signals measured by one detecting element, acquired over a time period of 250 s, with respect to the first acquired signal in that measurement.

Fig. 3 shows the results of the analysis conducted on data acquired with the detectors stationary over 250 s. The sample-shifts to align signals received by a particular detector element at different time points give an indication of movement of the breast over that time period. Fig. 3(a) shows an example of such an alignment, with the alignment performed for the most dominant transient in the signal trace, by shifting the first signal to maximize the correlation with the other signal (*s*).

Fig. 3(b) shows the required shift with respect to the first acquired signal, of all signals received by a particular element. This analysis was done for two subjects (1 and 4), where the situation without breast-supporting cup (shades of red) is compared to that with a cup (shades of blue). The total time period over which this analysis was conducted is 250 s, in which 2500 signals were received from illuminating the breast with 755 nm. On the *y*-axis, for each alignment, the shift is plotted in mm. A shift of one sample corresponds to 1 over the sampling frequency (25 MHz) multiplied by an SOS of 1500 m/s. It is immediately clear that the required shifts with use of a cup are zero or negligible (around 0.1 mm), and stable as the measurement proceeds. Without a cup, on the contrary, there are two effects playing a role. First, there is an overall increase in the required shift during the measurement period. Towards the end of the measurement period of four minutes, signals need to be shifted 1.5 or even 2 mm (for subjects 1 and 4, respectively). The fact that these signal shifts all have a positive sign, means that signals need to be shifted to arise later in time to align with the first signal, indicating that the breast comes closer to the detector over time. For subject 4, this would correspond with a slow sagging of the breast over the measurement period of four minutes, since the detector element is at the bottom of its array and thus facing upward in the imaging tank. The detector element chosen in the analysis of subject 1 is halfway the detector array, where it can be expected that vertical sagging of the breast also shortens the acoustic path length from breast to detector. The second effect that is visible in both subjects, is regular increase and decrease of the shift on a much shorter time scale, riding on the general shift increase. For subject 4, the period of these fluctuations is approximately 6 s. This could very well correspond to respiratory motion. For subject 1, the period is approximately 3–4 s. The amplitude of these fluctuations is higher in subject 4, while the time period of one cycle is longer, compared to subject 1. This might indicate a slow, but deep respiration by subject 4, and a quicker, but more superficial respiration by subject 1.

**Fig. 4:**
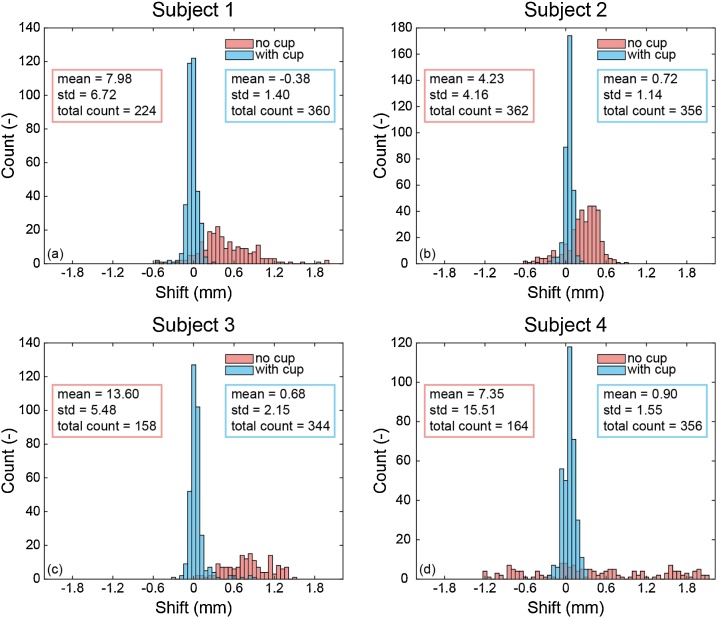
(a)–(d) The last signal (*t* = 250 s) was aligned with the first signal, for all detecting elements (384) in each measurement. The required shifts (in mm) to align the signals are plotted in histograms, each subject in a subfigure. A comparison was made between the measurement without and with breast-supporting cup. The boxes show the mean, standard deviation (std) and total count of aligned signals included in the analysis (out of 384 in total, after thresholds were applied).

Fig. 4(a) through (d) visualize the distribution of required signal shifts for aligning the last with the first signal, for subjects 1 through 4, respectively. For each subject, the measurement without cup is shown in red and the one with cup in blue. A red and blue box indicate the mean and standard deviation (std) of the shifts without and with cup, respectively. Signals acquired by all 384 single detecting elements were initially included in the analysis, but after applying thresholds, some of them were excluded. The total count numbers in the red and blue boxes indicate the number of elements of which signals were included for analysis (out of 384).

From the histograms it is clear that the distribution of required shifts is wider when no cup is used. The standard deviations are always higher in the measurement without a cup. This suggests that with the use of the cup, signals acquired after four minutes need less of a shift to be aligned with the first acquired signals, denoting less movement and thus an immobilized breast.

### 3.2 Image quality without and with support

**Fig. 5:**
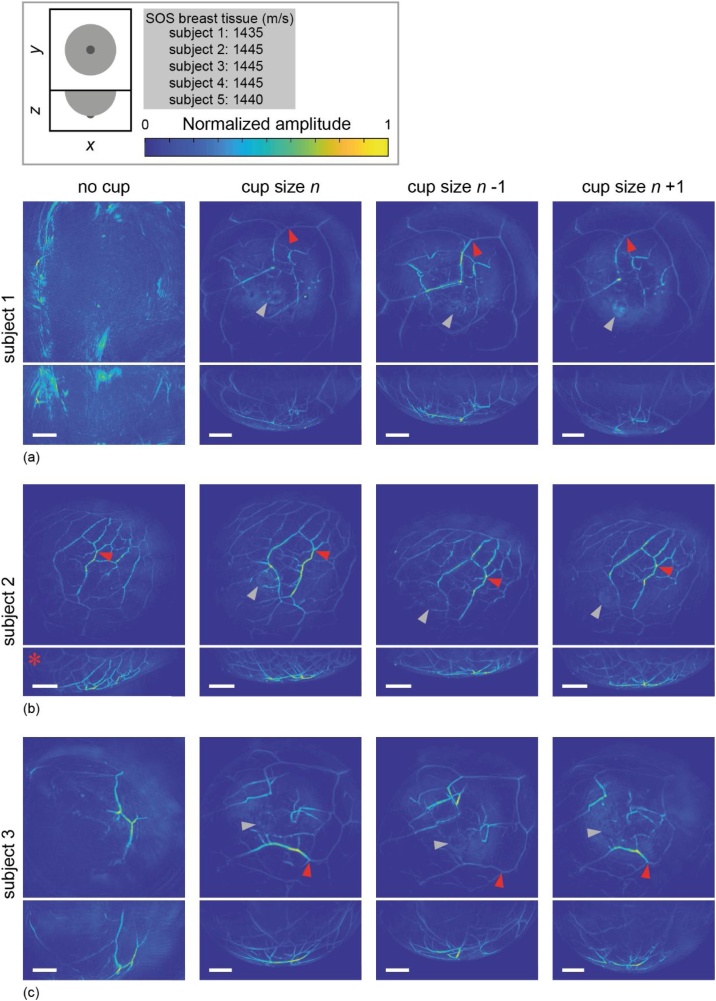

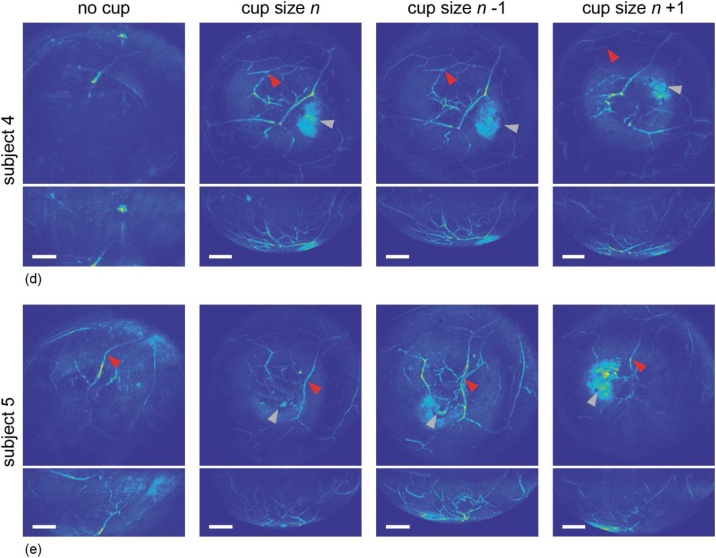
Reconstructed MIP images of five healthy volunteers ((a)–(e)), obtained by illuminating the breast with 755 nm light. The first column shows images without the use of a cup, columns 2 to 4 show images obtained with different sized cups, where the size is indicated above each column. For each subject and each measurement, a coronal view (*xy*) as well as the corresponding sagittal view (*xz*) are shown together. All scale bars represent 20 mm. For all subjects, red arrowheads indicate a recognizable bifurcation or vessel. Gray arrowheads indicate the (presumed) nipple location.

Fig. 5 shows reconstructed images (coronal and sagittal MIPs) from five healthy volunteers, imaged without breast support (column 1) and measured with three different sizes immobilizing cups (columns 2 through 4). All images shown here were obtained by illuminating the breast with 755 nm light. The SOS applied for reconstructing the part of the image inside the cup boundary is mentioned (per subject) in the top of the figure. In all images, red arrowheads indicate a specific bifurcation or vessel, that can be recognized in all cup images of that subject, in some cases, also in the non-cup image of that subject. Gray arrowheads indicate the nipple in the measurements with cups, or in some cases the presumed nipple, since it cannot always be clearly localized.

For all subjects, a clear difference can be seen comparing measurements with a cup versus those without a cup. For subjects 1 and 4 (Fig. 5(a) and (d), respectively), no typical breast shape is seen when the breast is freely pendant (column 1), nor any clearly reconstructed blood vessels. Columns 2 through 4 however, employing different sizes supporting cups, show blood vessels and in the case of subject 1, also the nipple (gray arrowheads). The high-intensity patches visible in all three cup measurements of subject 4 (gray arrowheads) might be attributable to the nipple, since it can be deduced from their locations with respect to neighboring vessels that they originate from the same portion of the breast. Moreover, from the sagittal images, we see that the patches belong to a superficial part of the breast.

The measurement on subject 2 (Fig. 5(b)) without cup already shows a breast contour and a detailed network of blood vessels. By extrapolating the breast contour however, we see that not the entire breast was in the measurement volume (see red * for the part of the breast that is already barely visible). More of the breast is visible in the measurements with cups (columns 2 through 4). Vessel morphology is very well comparable in all measurements of this subject; certain bifurcations and patterns are unambiguously recognizable in all images. The red arrowheads illustrate an example. The indicated bifurcation is, however, not always at the same location within the measurement volume. Apparently, the breast position is not the same in all three sizes of the cup. That same effect is evident in images of subject 3 (Fig. 5(c)), where again red arrowheads point out a bifurcation. Both in Fig. 5(b) and (c), the nipple is visible and indicated with a gray arrowhead. Its appearance is however not constant, as can be learned by comparing for example columns 3 and 4 from Fig. 5(b), subject 2. This might be explained by its position relative to the bottom illumination bundle (illuminating the nipple side of the breast from the bottom of the imaging tank); the more central its position, the more light it receives.

The images obtained from subject 5 (Fig. 5(e)) look rather different from each other. A few blood vessels and part of a breast contour are visible in the non-cup measurement (left column), but not very sharply. The measurement with cup size *n* – 1 (column 3) shows a detailed vessel network, of which most are also visible in the cup size *n* measurement (column 2), but less so in the *n* + 1 measurement (column 4). In column 3, the nipple can be localized (gray arrowhead); and from its relative position, we can localize it in column 2 and 4 as well (again, gray arrowheads), although with a different appearance. Especially in column 4 (the largest size cup), its appearance is patch-like, as in subject 4 (Fig. 5(d)).

As for the differences observed between measurements with varying cup sizes; it seems, in all subjects, that the largest size (*n* + 1, column 4) visualizes the least amount of vessels and details. Size *n* and *n* – 1 (columns 2 and 3) are more comparable, where in subjects 1, 3, 4 and 5 (Fig. 5(a), (c)–(e), respectively) we conclude that the smallest size (column 3) shows somewhat more detail. Only in subject 2 (Fig. 5(b)), the measurement with cup size *n* visualizes more structures, perhaps the breast was pushed upwards, outside the measurement volume, with the smallest cup.

All in all, for all five subjects, better images are obtained with the use of breast-supporting cups. This leads to the visualization of (more) blood vessels and structures, which are also more sharply delineated. The breast is automatically positioned in the center of the imaging tank, avoiding the situation where part of the breast is outside the measurement volume. The latter is the case in most measurements without a cup.

### 3.3 Application of 2-SOS model

**Fig. 6:**
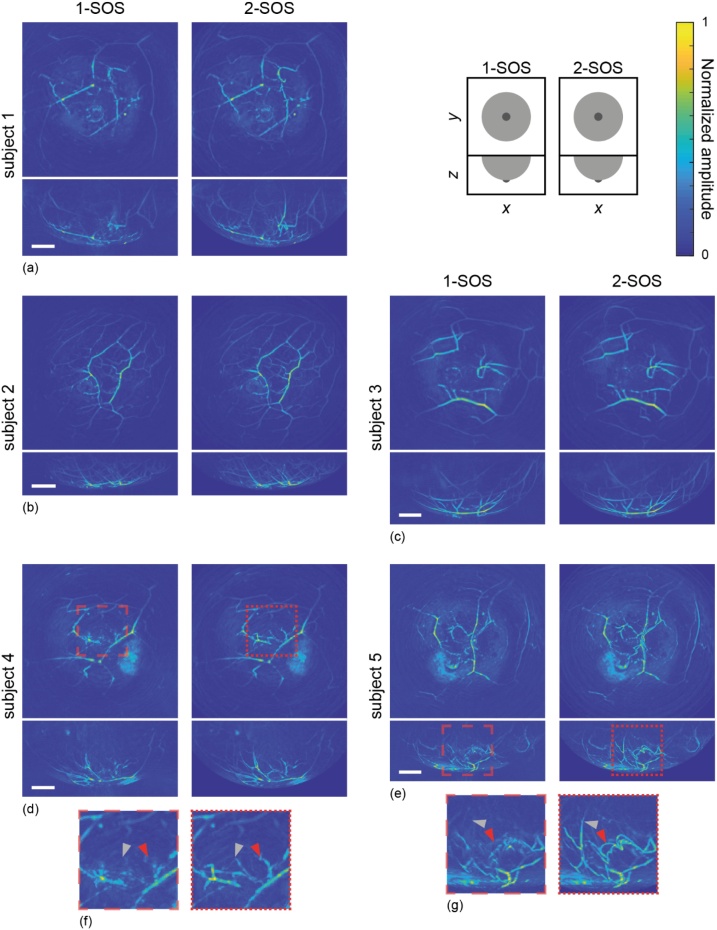
Comparison of 1-SOS and 2-SOS reconstruction algorithms. Reconstructed MIP images of five healthy volunteers ((a)–(e)), obtained by illuminating the breast with 755 nm light and by using a breast-supporting cup. For each subject, the left column shows images obtained with a 1-SOS reconstruction, and the right column shows images from the same measurement obtained with a 2-SOS reconstruction. For each measurement, a coronal view (*xy*) and the corresponding sagittal view (*xz*) are shown together. All scale bars represent 20 mm. (f) Zoomed subsections (200 %) of the 1-SOS reconstruction (left) and the 2-SOS reconstruction (right) of subject 4. (g) Zoomed subsections (200 %) of the 1-SOS reconstruction (left) and the 2-SOS reconstruction (right) of subject 5. Red and gray arrowhead pairs point to vessels more clearly resolved in the 2-SOS reconstructions compared to the 1-SOS reconstructions.

Creating a clear water-tissue boundary, required for a reconstruction with two speeds of sound (2-SOS), was another goal of the supporting cup. It can already be seen from columns 2 through 4 of Fig. 5 that indeed a contour is visible. The known contour of the cup was used for the reconstruction algorithm. To assess the added value of this 2-SOS reconstruction, measurements conducted with supporting cup were also reconstructed with just one SOS (1496 m/s), based on the temperature of the water in the imaging tank. The comparison of both reconstruction results for one measurement per subject can be seen in Fig. 6. It shows reconstructed images (coronal and sagittal MIPs) from all five healthy volunteers. For each subject, the left images show MIPs obtained with the 1-SOS reconstruction and the right images show MIPs obtained with the 2-SOS reconstruction. For subjects 4 and 5, subsections of the MIPs are shown enlarged (at 200 %) in Fig. 6(f) and (g) respectively. The left zoomed boxes show the 1-SOS reconstruction; the right boxes the 2-SOS reconstruction of the same volume.

In general, in all 2-SOS reconstructions, blood vessels are reconstructed with greater detail and more or longer blood vessels are visible. In the zoomed subsections of subjects 4 and 5 (Fig. 6(f) and (g), respectively), red and gray pairs of arrowheads point out clearly resolved vessels in the 2-SOS reconstructions, which are not or less resolved in the 1-SOS reconstructions.

### 3.4 Breast volume assessment

Table 3 shows the estimated breast volume per subject, when the breast is freely pendant inside the imaging tank filled with water, lying in a prone position. All volumes have been rounded to centiliters. The breast volume of subject 5 was not measured. Table 3 also lists the volumes of the three cups that were used for measurements on each subject.

**Table 3:** Estimation of volume of the breast when freely pendant in the imaging tank, without supporting cup. Volumes of supporting cups employed in measuring these subjects.

Subject	Volume of breast (mL)	Volumes of cups (mL)
1	1410	717, 856, 1013
2	430	264, 331, 408
3	1060	717, 856, 1013
4	1360	717, 856, 1013

The breast volumes of all four subjects are higher than the volumes supported by the cups that were used for measurements on these subjects. This suggests that not the entire breast was in the measurement volume when it is supported by a cup.

## 4 Discussion

In this paper, we have shown the design and added value of using breast-supporting cups for photoacoustic tomographic breast imaging. Through various experiments, it was demonstrated that the use of the cup reduces breast movement, leads to improved breast positioning in the field-of-view, and enables application of a 2-SOS model. This all contributes to the improvement of image quality, compared to a no-cup situation. All measurements in this paper were carried out on subjects above the age of 50, with relatively fatty breasts. The expectation is that the advantageous effect of the cups will be less in more dense breasts. This is partly based on our experience with measuring on young healthy subjects below the age of 30, as presented in Ref. [25]. The effect of the cups on measurements on dense breasts is however still to be investigated.

Movement analyses of the breast were performed with and without cup-support by acquiring 2500 consecutive photoacoustic signals over a time period of 250 s with stationary ultrasound detectors (Fig. 3, Fig. 4). The added value of the cup was demonstrated. Without a cup, the breast shifted over 1.5 or even 2 mm during the measurement period (Fig. 3(b)), which results in impaired images from tomographic scans. With the cup, breast movement is limited to ∼ 0.1 mm, which is well below the system resolution (∼ 1 mm, [25]). On top of the slow movement, apparently a sagging of the breast into the water, a repeating alternating movement within a time period of 3–6 s was observed. In normal breathing, the respiratory rate is around 20 per minute [37], which equates to one inspiration and expiration in 3 s. Slow, deep breathing occurs at a rate of 10 per minute, or 6 s for one cycle. These numbers suggest that breathing patterns were observed in Fig. 3(b), where the breathing pattern of subject 4 is more deep and slow compared to that of subject 1. This breathing motion is not visible when the cup is used. In a study conducted by Ruiter et al. [38], the movement of the breast in a 3D ultrasound computed tomography system was measured over eight minutes, where the subjects were in a comparable prone position with the breast in a water-filled aperture. They too reported on a slow directed movement (which they attribute to muscle relaxation) and some random or periodic movement, due to breathing. The measured mean distance between initial and final subject position was 2.2 mm, which is comparable to, but slightly higher than the movement we recorded in half the measurement time. From Fig. 3(a), we see that he required shift is not equal for the entire signal. The first high peak is aligned after shifting, but the second smaller peak is not aligned with this shift. This indicates a non-rigid movement of the soft tissue, or could be caused by the detectors receiving not only signals from structures on its acoustic axes. A rigid movement would then cause a variation in shifts for signals obtained from different absorbing structures, within the same signal. It would therefore be difficult to do a correction for movement in post-processing, and preventing movements using the cup is a better solution.

From studying required shifts of signals acquired by all 384 detector elements, histograms visualizing the distribution of shifts per measurement were obtained (Fig. 4(a)–(d)). The total count refers to the number of detectors that were included in the analysis, after applying thresholds to remove outliers. It is striking that for three out of four subjects (except subject 2), the total count of included signals is lower in the no-cup measurement than in the cup measurement. This is most likely an extra effect of the movement. Since we compare the last acquired signal with the first acquired one, which were acquired four minutes apart, it is difficult to align these signals with a high correlation coefficient when the breast has moved in between.

It is interesting to see the relation between the distribution of shifts, and the added value of the cup of the same subjects in reconstructions of a regular tomographic scan (Fig. 5). Subject 2 (Fig. 4(b)) shows the smallest spread in required shifts in the measurement without cup (std = 4.16) and has the highest total count of included signals for analysis, both indicating that this breast suffered the least from movement over four minutes. The maximum shift for this subject is 15 samples, equal to a movement of 0.9 mm. When looking at Fig. 5(b), column 1, showing reconstructions of the same subject, it becomes clear that indeed a measurement without breast support already led to an image where breast contour and a vascular network are visible. This is likely related to the breast size of this volunteer (see Table 3). Subject 2 had a breast volume of ∼ 400 mL, whereas the other four subjects had breast volumes above 1000 mL. It is plausible that a larger breast volume moves more, distorting the image quality in a four-minute scan. When looking at subject 4, we see the largest spread in required shifts (Fig. 4(d)) when not using a cup (std = 15.51). Indeed, for this subject, the improvement in reconstructed images by using a cup was significant (Fig. 5(d)). Without the cup, we could not recognize a breast shape and only part of a vessel was vaguely reconstructed. Out of the four subjects for whom the movement analysis was done, subject 3 has (after subject 2), the smallest breast (breast volume of ∼ 1000 mL compared to >1300 mL for subjects 1 and 4, see Table 3). In the movement analysis, the spread in required shifts is again, after subject 2, the smallest (Fig. 4(c)). The reconstruction of a measurement without cup (Fig. 5(c), column 1), already shows some vascularity and part of a breast contour, more so than subject 1 and 4 (Fig. 5(a) and (d), respectively). We are therefore confident to conclude that the smaller the breast, the smaller the movement when using no support, and the smaller the improvement in image quality when supporting the breast. However, for small breasts, the advantage of positioning the breast in the center of the imaging tank with the cup is still evident.

This work also shows that the use of a cup allows optimal positioning of the breast in the field-of-view of the detecting aperture. It can be seen from Fig. 5, the first column, that without breast support, the breast was not in the center and thus not entirely in the measurement volume. This is particularly clear in Fig. 5(b), because in this measurement the breast contour is obvious also without cup. Only the superior part of the breast is in the measurement volume here. Something similar is visible in Fig. 5(e). The vessel marked with the red arrowhead serves as a landmark from which it can be deduced that again the superior portion of the breast is in view. For all subjects, columns 2 through 4 show that the cup forces the breast to the center of the imaging tank, indeed leading to a larger portion of the breast being imaged.

Although the cup positions the breast in the center of the imaging tank, there is still some variation in positioning of the breast within the cup. This is illustrated by red arrowheads in Fig. 5, indicating recognizable vessels or bifurcations in all images. For all subjects, these landmarks are not in the same position in each image, indicating different locations of the breast. Since columns 2 through 4 show measurements with different sized cups (three successive sizes for each subject), it is plausible that the size of the cup plays a role in the position of the breast, together with the fact that the breast is composed of soft tissue. We do not foresee any problems with this, since the entire breast is always in the measurement volume. This positioning variability does however make it difficult to perform quantitative quality assessment based on selection of a region of interest (ROI). Depending on the location of a ROI within the imaging volume, it might receive more or less light and thus be reconstructed with higher or lower contrast, respectively. This difference would not be necessarily related to the improvement caused by using a certain size cup. We can qualitatively perceive these differences in contrast within an image. For example in Fig. 5(d), cup size *n* visualizes the vessel depicted by the red arrow with high contrast. Other vessels however seem to have a higher contrast with size *n* – 1. But, on the other hand, if we were to assess image quality of the entire image, structures such as the highly absorbing patches in Fig. 5(e) might contribute positively to the contrast while not necessarily leading to the most informative image.

In earlier measurements with PAM 2, one SOS was used for reconstruction of the entire measurement volume, based on the temperature of the acoustic coupling water. Assuming a water temperature between 24 °C and 26 °C (goal temperature of 25 °C), the 1-SOS reconstructions typically used values between 1494 and 1499 m/s [36]. Oraevsky et al. [23] used a reconstruction based on Ref. [39], employing a constant SOS throughout the measurement volume. The value of the used SOS is not stated. Lin et al. [22] refer to a half-time algorithm described in Ref. [40], where artifacts due to heterogeneous acoustic properties are mitigated by utilizing the first-half data function. Matsumoto et al. [24] described a reconstruction where the SOS was categorized into two layers, based on the water temperature difference below and above the supporting cup. Deán-Ben et al. [41] showed the value of considering different SOS for tissue and coupling medium. Moreover, it is known that the SOS of breast tissue can greatly vary [[42], [43], [44], [45]], depending on the distribution of tissue types. Therefore, the choice was made to not only employ a 2-SOS model, but also tune the tissue SOS per subject. Fat typically has a lower SOS than fibroglandular tissue. Literature values for fatty breast tissue typically lie around 1440 m/s. All our subjects were above the age of 50 at the time of measurement, so presumably have relatively fatty breasts. This is indeed confirmed by the empirically optimized SOS employed in our subjects (all between 1435 and 1445 m/s). It would be interesting to do 2-SOS reconstructions in younger subjects, to assess whether their breasts, having a higher amount of fibroglandular compare to fatty tissue, indeed have a higher SOS. It is expected that obtaining an SOS-map of the breast, taking into account the distribution of different tissue types, could further enhance image quality when these SOS are applied in the reconstruction. In empirically choosing tissue SOS in the current study, different regions of the breast were reconstructed with detail when altering the SOS. Given the generic and rotationally symmetric shape of the cup, it can be expected that in some cases, the cup will not perfectly follow the breast contour. We do not foresee problems with this in applying the 2-SOS model, assuming the deviation of the tissue contour with respect to the cup contour is small. The 2-SOS model will still give a considerable improvement compared to applying only one SOS for the entire volume. Moreover, since we have eight sizes, we can seek for the best fitting option, leaving as little space as possible between breast and cup while supporting the breast.

We also studied the effect of multiple sizes of breast support. Our results show that a small cup size is favorable for multiple reasons. First of all, the thickness of the tissue through which the light (from the bottom illumination, in our case) needs to penetrate is small. As can be seen for example in Fig. 5(d), column 4 (measurement with cup size 8), there is a portion illuminated by the bottom illumination, and a portion of the breast illuminated by the side illumination beams. In between these regions, less light has reached the tissue, creating a kind of shadow in the photoacoustic signal. The smaller the cup, the higher it is in the imaging tank, the larger the spot of the (divergent) bottom illumination is. The area illuminated by the bottom beam was estimated to be twice as big when using the smallest cup compared to using the largest cup, when illuminating with 755 nm. This means that, while a larger area is illuminated when using a smaller cup, the fluence on this area is lower, potentially altering contrast. Since we do not make any quantitative statements about exact reconstructed pressures, this does not pose a problem here. When in future measurements we want to assess contributions of chromophores, all measurements on a subject will be performed with the same cup size, eliminating this issue. Although the illuminated area is larger when using a smaller cup, too small a cup would most likely push the breast towards the chest wall, not having the portion of the breast close to the chest wall in the measurement volume. Our breast volume measurements confirm this. For example, subject 1 had a breast volume of ∼ 1400 mL, and was measured with cups in sizes 6 through 8. Cup 6 only has a volume of 717 mL, almost half of the breast volume of the subject. Cup size 8, having a volume of 1013 mL, is also still smaller than the subject’s breast volume. This suggests that a portion of the breast was not in the cups (all three sizes) and was therefore not measured. There is obviously a trade-off between stability and anatomical availability. It has to be researched, in clinical measurements on breast cancer patients, whether tumors close to the chest wall and/or in the axillary tail of the breast are in the measurement volume when the breast is pushed upwards.

The PVC material used here could potentially be used for other applications in photoacoustic imaging, such as preclinical animal imaging. The purpose would be to position and hold the sedated animal at a fixed position in the imaging volume, without adding too much extra material that can potentially distort or obscure the image. By 3D-printing a mold in a desired shape, holders can be tailor-made. For other imaging systems, it would however be advisable to characterize optical and acoustic transmission for the applicable illumination wavelengths and acoustic frequencies.

## 5 Conclusion

All in all, we have given an elaborate description of the development of breast-supporting cups for photoacoustic tomographic breast imaging. We showed a comparison between measurements without and with support, clarifying the added value. We have demonstrated, by various experiments, that the cup positions the breast in the center and thus the measurement volume of the system, reduces movement caused by sagging of the breast but also by breathing, and enables a more detailed reconstruction through a 2-SOS model. All these factors contribute to a higher image quality. Moreover, multiple (eight) sizes of these cups have been developed, accommodating a wide range of breast sizes.

## Declaration of Competing Interest

Authors T. J. P. M. o. R., R. P. P. v. M. and L. A. are employed by PA Imaging R&D B.V. but have no financial interest in the company. The authors declare that there are no other conflicts of interest.
